# Dr. William Tod Helmuth

**Published:** 1902-06

**Authors:** 


Dr. William Tod Helmuth, of New York, head surgeon of the Flower Hospital, and one of the foremost representatives of the homeopathic school of medicine in this country, died at his home, No. 67 Madison Ave., from heart disease May 15, 1902. He was born in Philadelphia on October 30, 1833, was a graduate of the Homeopathic Medical College, Philadelphia, in 1853 and in 1888 received the degree of LL.D., from Yale University.

He was ex-president of the American Institute of Homeopathy, of the New York State Homeopathic Medical Society, and the New York County Homeopathic Society. He was also president of the Collins State Homeopathic Hospital and an honorary member of the Société Medicale Homeopathique of France.

